# Cervical Artery Dissections—A Demographical Analysis of Risk Factors, Clinical Characteristics Treatment Procedures, and Outcomes—A Single Centre Study of 54 Consecutive Cases

**DOI:** 10.3390/jpm14010048

**Published:** 2023-12-29

**Authors:** Iulian Roman Filip, Valentin Morosanu, Doina Spinu, Claudiu Motoc, Zoltan Bajko, Emanuela Sarmasan, Corina Roman, Rodica Balasa

**Affiliations:** 1 Department of Neurology, “George Emil Palade” University of Medicine, Pharmacy, Sciences and Technology, 540136 Targu Mures, Romania; roman-filip.iulian.22@stud.umfst.ro (I.R.F.); morosanu.valentin.23@stud.umfst.ro (V.M.); spinu.doina.21@stud.umfst.ro (D.S.); mclau1996@yahoo.com (C.M.); sarmasan.emanuela.22@stud.umfst.ro (E.S.); rodica.balasa@umfst.ro (R.B.); 2 Department of Neurology, Faculty of Medicine, “Lucian Blaga” University of Sibiu, 550169 Sibiu, Romania; corinaromanf@ulbsibiu.ro

**Keywords:** cervical artery, dissection, stroke, compression symptoms, conservative treatment, endovascular treatment, prognosis

## Abstract

Cervical artery dissections (CAD) are a common cause of ischemic cerebrovascular events among the younger and middle-aged population. Altogether, CAD counts for up to 15% of all causes of stroke in patients aged 50 or younger. Among the known etiological causes, especially addressing the younger population with mechanical traumas and whiplash injuries are regarded as the main culprits. However, cases of spontaneous dissection are also widespread, with risk factors such as hypertension, migraine, and lifestyle factors increasing the risk of occurrence. Clinically, the symptoms associated with a cerebrovascular event caused by CADs are highly variable and can be classified as either compressive symptoms (such as Horner’s syndrome and cervical pain) or stroke syndromes attributable to cerebral ischemia. Therefore, establishing an early diagnosis might be particularly challenging as it requires particular attention and quick clinical reasoning when interviewing the patient. With these certain particularities, our main focus was to conduct a prospective study involving up to 54 patients who were diagnosed with CAD in our clinical facility between January 2015 and December 2022, with the focus of assessing certain individual parameters attributable to each patient and their influence and prognosis value for their short and long term evolution. An important emphasis was placed on parameters such as topographical localization, clinical presentation, severity of the questioned cerebrovascular event, outcomes, and causative factors. Statistical validity tools were applied when possible.

## 1. Introduction

Cervical artery dissection (CAD) is a complex term that refers to both carotid artery dissections and vertebral artery dissections. An arterial dissection is the direct result of a longitudinal injury in the arterial wall intima or the vasa vasorum, leading to an intramural hematoma, which can separate the vessel wall, creating a false lumen, leading to local compression or vessel occlusion and causing ischemia. CAD is generally attributable to the younger population with a particular set of predisposing factors such as genetic pathologies, injuries caused by trauma and neck strain, and diseases that influence clotting and platelet function. Gender can also influence the outcome for those patients [1,2].

The various clinical syndromes that can be linked to cervical artery dissection (CAD) are complex and attributable to various independent factors. 

CAD is responsible for as much as 25% of ischemic strokes in patients aged 45 years or younger. The most commonly incriminated vessel is the internal carotid artery, followed by the vertebral artery and the common carotid artery, with incidences ranging from 1 to 3/100,000 people [2,3].

The cause of CAD is either spontaneous or traumatic, with the pathogenetic cause of its occurrence being highly debatable.

Cervical strain or trauma is a widely known culprit, as even small-scale trauma related to usual household or workplace activities can trigger the symptoms and is not unusually correlated with several predisposing conditions for vessel friability. Some of those conditions include fibromuscular dysplasia and connective tissue disorders such as Ehlers–Danlos syndrome or Marfan syndrome.

The dissection that occurs suddenly without an underlying traumatic insult is called spontaneous. Risk factors such as hypertension, migraine, and several habitual factors have been consistently associated with spontaneous dissections. The male population is often associated with this pathogenic sequence [4].

Patients with CAD can either remain asymptomatic or slightly asymptomatic, with cervical pain remaining their sole symptom. Those dissections are generally attributable to compressive causes. CAD-causing ischemia can produce symptoms related to the vascular territory involved. In the case of internal carotid dissections, compressive syndromes, such as neck pain, Horner’s syndrome, or hemicrania, precede the onset of the stroke syndrome by days in some cases, while those attributable to the posterior circulation can potentially mimic a distinct medical problem, leading to diagnostic challenges (with dizziness and posterior cervical pain usually suggestive of cervical spondylosis) [5,6].

CADs usually carry on a promising prognosis, with spontaneous healing generally occurring within up to 12 months of symptom onset. 

There is, however, a relative scarcity of literary data regarding the influence of predisposing factors, traumatic factors, and even gender in the emergence and potential outcomes of stroke or compression syndromes attributed to CAD. In that regard, we conducted a prospective study of 54 distinct CAD cases that benefited from comprehensive care in a tertiary neurology clinic.

Our purpose was to analyze several demographical and prognosis data on relatively uniform racial distribution and to highlight distinct factors contributing to general outcomes in the Eastern European population, as there is a limited amount of data in this particular direction.

## 2. Materials and Methods

We conducted a prospective, observational study involving 54 consecutive cases of CAD that have been admitted and treated at a tertiary neurology clinic in Targu Mures, Romania, between January 2015 and January 2023. These cases represented up to 0.43% of all stroke cases that were hospitalized in our department. CAD cases were included in our stroke database following their discharge, and their subsequent evolution was evaluated at telephonic follow-ups when possible.

The patients were included in this study based on the following perquisites:

- Age >18 years;
- Certain diagnoses of CAD using conventional and valid diagnostic methods;
- New onset of symptoms before diagnosis without previous knowledge of dissection history;
- The compliance of the patient, both in the short and in the long terms;

The cases that did not meet those criteria were not included in our study.

The methods of diagnosis were via computer tomography (CT), computer tomography arterial angiography (CTA), direct subtraction angiography (DSA), magnetic resonance imaging (MRI), and Doppler ultrasonography.

After thorough investigations, several important data were obtained from the patients, including a comprehensive risk factor assessment, clinical characteristics, and eventual outcomes. The clinical severity of the stroke associated with CAD was further assessed using the National Institute of Health Stroke Scale (NIHSS) at admission, while we assessed the discharge status using the modified Rankin scale (mRS) at discharge. When possible, the clinical status of the patients was evaluated at follow-ups using the modified Rankin scale.

Ethical approval was also obtained from the institutional review board of the hospital (07.03.2023).

Several descriptive methods, such as percentages, frequencies, means, and standard deviations, were used to perform the statistical analysis. Furthermore, the patients were distributed in groups to which we applied the Kolmogorov test for continuous variables. The data were subsequently analyzed using a two-sample *t*-test and the Mann–Whitney U test depending on the result of the Kolmogorov formula. The association between categorical factors was assessed using Fisher’s exact test, chi-square test, and Spearman’s test, depending on the variable. When comparing more than two groups, a one-way ANOVA test was performed for parametrically distributed data, and the Krutschal–Wallis’s test, followed by the Dunn test, was performed. For the data that were interpreted as parametric, the mean value was calculated, while for the data that were interpreted as non-parametric, the median values were provided depending on the particular situation. The interquartile range was calculated when possible for the parametric data.

For linear regression, considering alpha to be statistically significant at 0.05 hallmark, the *R*-value will suggest a strong correlation between variables at a higher value than 0.7.

A comparison was made between male and female patient groups, with a further subdivision into male subgroups, with risk-underlying risk factors, such as hypertension, neck strain or trauma, blood clotting issues, or environmental risk factors, such as smoking and alcohol consumption.

Overall, the following parameters were studied:

- The age profile of the patients;
- The underlying mechanism of the dissection;
- The impact of the risk factors on the general prognosis of the subjects;
- The mean morbidity of the patients upon discharge;
- The clinical presentation.

There are several types of statistical inaccuracies or errors that are predictable and have been regarded by the authors, such as the following:

- Sample errors were given by certain anomalies in the distribution of the studied population. We managed to avoid this type of error by focusing on a relatively homogenous demographical group;
- Use of a relatively small data sample, given the relative scarcity of cases and the rarity of the disease, further application of the statistical method that was used should be further implemented on larger populations.

## 3. Results

As mentioned above, 54 patients were included in this study, with 18 being female (34%) and 36 being male (66%) (*p* = 0.2). Women were younger than men (*p* = 0.04), as the mean age of the female group was calculated at 37.7 ± 2.37, while the mean age of the male group was reported at 41.6 ± 1.83.

There was a relatively equal ethnical distribution of those patients, as all were of Caucasian descent.

### 3.1. Clinical Presentation Associated with Risk Factors and Diagnosis Tools

The mean NIHSS clinical score of the patients diagnosed with stroke following CAD was 6 ± 0.78. No significant difference was found between the presenting NIHSS scores of the male patients and the female patients (*p* = 0.6).

On one hand, neck strain following strenuous physical exercise, Valsalva maneuvers, or direct cervical trauma were reported in as many as 19 patients involved in our study; the mean NIHSS score of those patients was reported at 6.24 ± 1.49. On the other hand, 31 patients presented with cervical artery dissection without any history of the above-mentioned traumas, with a mean NIHSS score of severity at presentation of 5.9 ± 0.96, showing no significant correlation between cervical traumatic events and more severe presentation symptoms (*p* = 0.8).

The most common presentation symptom for our cohort was limb weakness (hemiparesis) (*n* = 38), accounting for about 76% of the patients; other common presenting symptoms were speech disturbances and aphasia (*n* = 12), accounting for about 24% of the cohort, headache, and neck pain (*n* = 10), accounting for about 20% of the patients in the cohort. Other less common symptoms included dizziness and vertigo (*n* = 5) in 10% of the patients, Horner’s syndrome (*n* = 3)—6%, diplopia, and cranial nerve palsies (*n* = 2) in 4% of the patients. Two patients presented only local symptoms without underlying stroke, and they were diagnosed using ultrasonographic methods.

**Figure 1:**
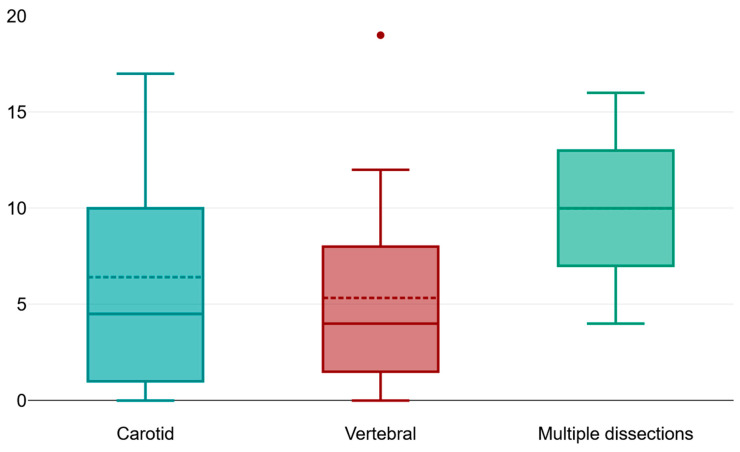
Box plot comparing the mean values of the clinical severity score NIHSS at admission with the incriminated vessel dissection.

There appears to be no significant statistical correlation between the number of affected vascular territories and the sex of the patients (*p* = 0.42) (Figure 1).

Several risk factors were significantly associated with worse clinical presentation symptoms, such as chronic alcohol consumption—(*p* = 0.004), with a mean NIHSS score at presentation for those patients of 10.1, while the non-alcoholic patients had a mean NIHSS presentation score of 5.43.

Older patients who were diagnosed with stroke caused by cervical artery dissection were more likely to be hypertensive with statistical significance (*p* = 0.001), with the mean age of the non-hypertensive patients being 34.75 ± 1.92 and 44.27 ± 1.85 for the hypertensive patients, respectively (Table 1).

Neither smokers (*p* = 0.5) nor hypertensive patients (*p* = 0.2) were associated with worse presenting symptoms, with the mean clinical severity score of smokers and non-smokers at 6 ± 0.873 and ± 1.85 and with 5.2 ± 1.022 as a mean score for non-hypertensive and 6.53 ± 1.12 for hypertensive patients, respectively.

Men were less likely to be suffering from migraines (12% of all men—*n* = 4), *p* < 0.006) than women (24% of all women—*n* = 4).

Prothrombotic conditions do not appear to significantly influence the clinical severity of the subjects (*p* = 0.5), with the mean NIHSS value for patients with underlying clotting conditions reported at 6.7 ± 1.41 and 5.63 ± 0.95 for patients with no apparent clotting issues.

Of 54 patients, 25 had hypercholesterolemia, totaling 46% of all patients, while 29 had no hypercholesterolemia. Of those with hypercholesterolemia, 16 were male (30% of the total patients and 65% of all patients with hypercholesterolemia), while 9 were female (16% of all patients and 34% of all patients without hypercholesterolemia).

There is a valid correlation between the patients with hypercholesterolemia and a worse presentation NIHSS score at admission (*p* < 0.001).

**Figure 2:**
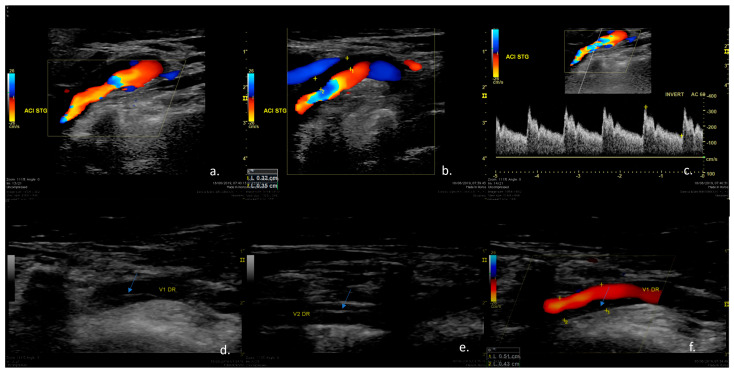
The Case of a 38-year -old marathon runner who presented during a competition a short episode of amaurosis fugax in the left eye and transient speech difficulties associated with cervical pain. Duplex ultrasound examination of the cervical vessels. (**a**,**b**) Color-mode examination revealing irregular stenosis at the level of the left ICA caused by hypoechoic vessel wall changes suggestive of mural hematoma. (**c**) Triplex-mode examination revealing increased blood flow velocities in the left ICA, suggesting severe stenosis. (**d**,**e**) B-mode examination of the right vertebral artery showing a hyperechoic line inside the vessel lumen (arrow), suggestive of intimal flap. (**f**) Color-mode examination of the right vertebral artery showing irregular stenosis caused by a hypoechoic mural hematoma (arrow). Therefore, the patient was diagnosed with two simultaneous cervical artery dissections at the level of the left carotid artery and the level of the right vertebral artery.

**Figure 3:**
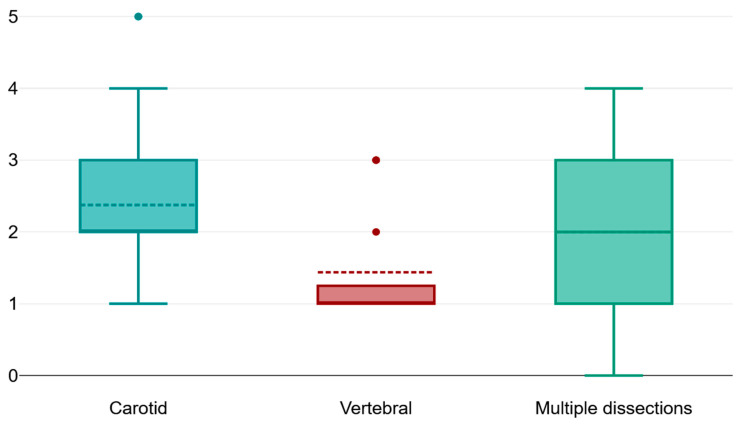
Box plot comparing the morbidity of the patients at discharge for each particular type of vessel dissection.

**Figure 4:**
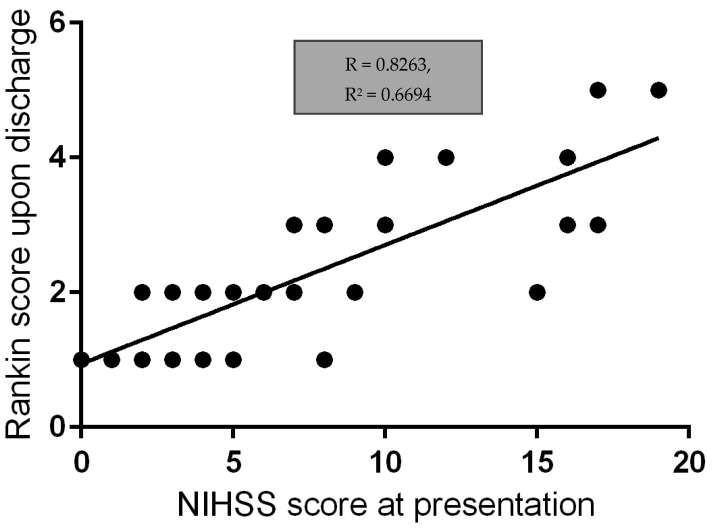
Linear regression correlation between NIHSS score at presentation and Rankin score upon discharge. The *R*-value suggests a very strong correlation between the NIHSS score at presentation and the Rankin score upon discharge, therefore implying that the clinical severity of the cerebrovascular event caused by CAD for our patients significantly impacts the outcome upon discharge.

**Figure 5:**
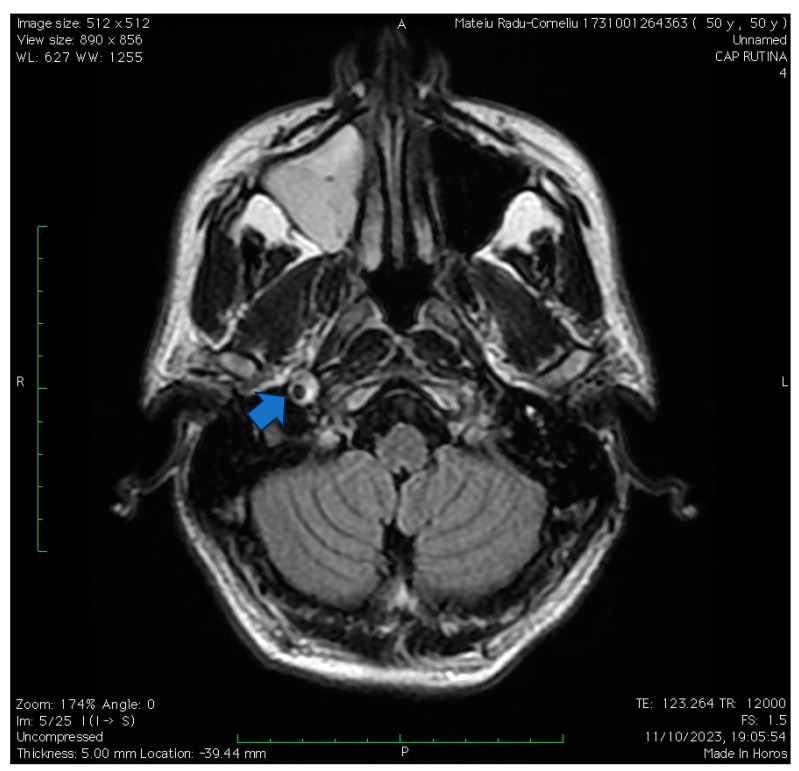
The case of a 50-year-old hypertensive and smoker male complaining of a weeklong anterior cervical pain and soreness following low-intensity physical effort. Upon clinical evaluation, the patient presented a right-sided Horner’s syndrome without other neurological signs. An angioCT exam and native CT scan were first performed, showing a unilateral mild right carotid artery narrowing in the immediate vicinity of the petrosal part of the temporal bone (corresponding to the proximal C3 segment of the carotid artery). A subsequent native MRI sign was performed and managed to highlight an intramural hematoma at the same level corresponding to the crescent sign (visualized using FLAIR sequence in this case- as pointed by the arrow). The ultrasonographic exam was non-remarkable. Anticoagulant therapy, as well as antiplatelet therapy, was initiated with slight improvement in regard to Horner’s syndrome upon discharge. Upon discharge, the patient received antiplatelet monotherapy. At a one-month follow-up, the patient remains oligosymptomatic, with a mild right-sided syndrome with normal pupillary function.

**Figure 6:**
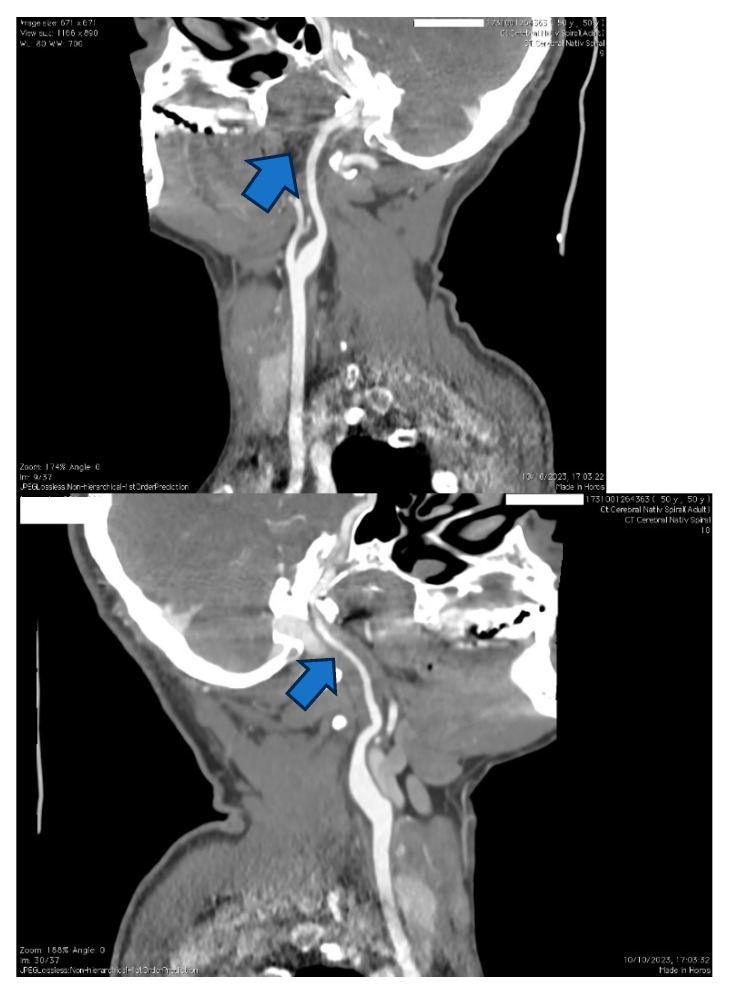
The same case of the 50-year-old patient, where a CTA was performed, showing focal tightening of the right carotid artery on C2 and C3 segments compared to the otherwise healthy left carotid artery.

**Figure 7:**
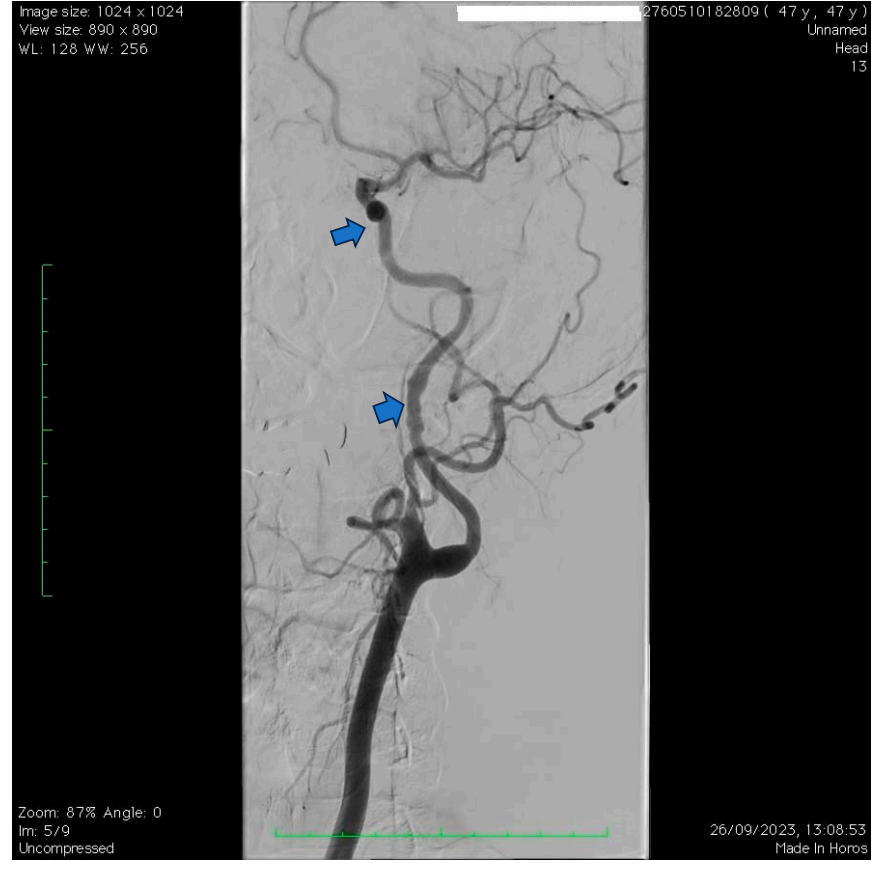
A case of a 47-year-old female with multiple documented cervical artery dissections who was referred to our center for a routine follow-up direct subtraction angiography. The angiography highlights typical focal widenings of the wall of the internal carotid artery in its rostral portion, as well as a pseudoaneurysm located within the cavernous portion of the artery. The findings are suggestive of fibromuscular dysplasia. Unfortunately, no previous angiography was available.

The most common diagnosis tool was via ultrasound methods (Figure 2) (Doppler ultrasonography) (*n* = 30) in 60% of the cases, with other methods, such as DSA (*n* = 10), in 20% of the cases and CT angiography in the remaining 10% of the cases (Figure 2, Figure 3, Figure 4, Figure 5, Figure 6 and Figure 7).

There is a very strong correlation between the NIHSS score at presentation and the Rankin score upon discharge, therefore implying that the clinical severity of the cerebrovascular event caused by CAD for our patients significantly impacts the outcome upon discharge (Figure 1, Figure 3 and Figure 4).

### 3.2. Short-Term and Long-Term Treatment Options and Outcomes

A total of six patients (12%) received endovascular treatment after receiving IV thrombolytic therapy. The number of patients who benefited from thrombolytic therapy was 10 (20%). The remainder of the patients received either anticoagulant or antiplatelet therapy during the acute phase (Table 2).

Regarding the long-term treatment of the patients, most of them (*n* = 25–50%) received anticoagulation with either antivitamin K or DOAC (direct oral anticoagulant) drugs, whilst the remainder 25 received two antiaggregant drugs (*n* = 13, 26%) and antiaggregant monotherapy (*n* = 12, 24%) (Table 3).

As per long-term treatment, most of the patients received anticoagulant treatment—25, double antiaggregant therapy—15, and mono antiaggregant therapy—15 patients. Five patients had thrombophilia. There were also two patients with Marfan’s syndrome.

The outcome of the patients was measured using the modified Rankin score, with the mean Rankin score of the patients being 1.85.

Women appear to have a better outcome reported by the Rankin score (*p* = 0.01), with the mean value of the Rankin score following discharge reported at 1.41 ± 0.17 in the case of women and 2.30 ± 0.22 in the case of men. Table 4—Comparison of mean Rankin scores at discharge and gender. The patient group was divided into two subgroups according to gender. The number of patients with a given Rankin score was counted. Lastly, the mean value of the Rankin score for the male and the female subgroups was calculated, and they were compared. The male subgroup had a significantly higher mean Rankin score upon discharge.

## 4. Discussion

The observational study conducted in our tertiary care clinic enlisted subjects over a period of almost ten years and managed to highlight a certain number of differences concerning the cervical artery dissection risk factor profiles and their influence on the severity of the clinical characteristics, as well as their influence on the discharge outcome. Even though the male subjects appeared to be more numerous, the mean age of the female patients diagnosed with CAD was slightly lower. For most of the risk factors, there is a significant male predominance that is attributable to lifestyle, such as hypertension, heavy alcohol consumption, or neck injury. This increased presence of risk factors in the male subjects persists even in the case of non-modifiable risk factors, such as coagulopathies. By analyzing the two subgroups, there appears to be no statistically significant predisposition for a risk factor in either group except in the case of the patients with a confirmed diagnosis of migraines, who were mostly female. The presence of certain risk factors appears to influence the clinical severity of the cerebrovascular event caused by CAD. This study managed to underline the association between risk factors, such as hypercholesterolemia and heavy alcohol consumption, and a worse clinical outcome. Those patients are naturally expected to have a worse long-term outcome. The general short-term outcome was quantified using the modified Rankin score, underlining a significantly better short-term outcome in female patients compared with male patients.

There is a number of conditions that have been previously studied and associated with a higher cardiovascular event occurrence rate. Those conditions can either be genetic, as in the case of Marfan’s syndrome, Ehlers–Danlos syndrome, fibromuscular dysplasia (genetic diseases that affect the natural architecture of the vessel), genetic diseases that affect the platelet function or general clotting (various forms of thrombophilia and hemophilia conditions), or several acquired diseases, as in the case of hypertension, acquired lipid metabolism disorders, or various types of cranial algic syndromes. We managed to highlight the occurrence of the cervical artery dissection associated with Ehlers–Danlos syndrome in one case, fibromuscular dysplasia in one angiographically confirmed case, and various types of thrombophilia in up to three cases.

As CAD is generally attributable to a younger age, there are several literature studies conducted previously with various results. A study conducted by Reges and his collaborators in 2019 was for a mean age of 44.5 years [7]. Another study conducted by Arnold M. and his collaborators managed to highlight a mean age of 47.5 years for male patients and 42.5 years for female patients [8]. Another study conducted by Smith and collaborators, whose main focus was on comparing vertebral artery dissections and carotid artery dissections, managed to find out that the mean age of those patients was 42.6 years in the case of vertebral artery dissections and 43.6 in the case of carotid artery dissections [9]. For our study, the mean age of our patients was identical in both subgroups at 41.6 years. Our results are generally close to the results that have been collected by other literature studies.

Our study also pointed out the slight male prevalence in the case of CAD, with 66% of the patients being male. This particular result is attributable to modifiable and non-modifiable risk factors. The retrospective study conducted by Mesto and his collaborators on a large cohort of 642 participants managed to also highlight a higher male prevalence, with 56.7% of the subjects being male [10]. Another study conducted by Audrey et al. further suggested male prevalence, with 61% of the participants being male. A higher male gender prevalence was also reported in a study conducted on 459 patients, setting the male prevalence at 52.9% [11]. Our results correspond with the data that were collected in other studies, therefore implying a higher male prevalence in the CAD population. Moreover, some rarer genetic and metabolical conditions usually associated with cervical artery dissections were not reported in our patients.

Several factors are associated with an increased gravity of symptoms upon presentation. Thus, risk factors such as dyslipidemia and chronic alcohol consumption were shown to have a significant impact on the severity of the stroke caused by CAD in our study. Indeed, the literature also strongly suggested the fact that there is a strong association between CAD and the mentioned risk factors. A multinational study carried out on 690 consecutive patients has shown a significant correlation between risk factors, such as dyslipidemia and increased BMI, and the occurrence of CAD but without a significant correlation between smoking, hypertension, or diabetes and this type of cerebrovascular event, showing similar results with the study that we have conducted [12].

Interestingly, some classically proven risk factors for cerebrovascular disease appear to have little effect on the clinical severity of stroke caused by CAD. Thus, risk factors such as hypertension, smoking, or prothrombotic conditions are not significantly associated with CAD and appear to have a nonsignificant impact on clinical impact. This can point out to a distinctive pathophysiological sequence in CAD rather than usual cerebrovascular events.

Women diagnosed with CAD are significantly likelier to have migraine, as the results of our study have pointed out.

Migraine is a multifactorial condition that has a distinctive pathophysiology. Migraine has also been previously associated with some serious conditions, including CAD [13]. Furthermore, strong evidence suggests the fact that disturbances in the trigeminovascular system caused by various factors are strongly correlated with this pathological entity. Thus, disturbances in cerebral blood flow play an important role both in CAD and migraine. The female gender has consistently shown a stronger predisposition toward migraine than their male counterparts [14]. Most commonly, metabolic factors and hormonal imbalances are likely to be the cause for a more significant female prevalence [15,16,17]. There is strong literature evidence that also links a more likely migraine predisposition in the case of women diagnosed with CAD rather than men. Indeed, the metanalysis performed by Daghals I and his collaborators revealed the fact that migraines are consistently associated with various types of stroke, including CAD. The same study also managed to suggest that several genetic factors are also likely to be correlated with the triad of migraine, stroke, and cervical artery dissection [18]. Another meta-analysis suggested the fact that migrainous subjects are two times likelier to develop CAD, regardless of the type of migraine or gender [19]. The Italian project of Stroke in Young People was a large cohort study whose purpose was to find potential links between migraine and cervical artery dissection. The study involved 2485 patients diagnosed with stroke as their first cerebrovascular event. After comparing the statistical results, the authors managed to find the following significant correlations: firstly, between CAD and migraine; and secondly, between female gender and migraine in patients diagnosed with CAD [20].

Our statistically acquired data managed to point out the fact that not only is migraine a significant risk factor for cervical artery dissection, but it is also related to a worse clinical presentation. Secondly, we have discovered that women with CAD are also likelier to have migraines than men with the same condition. These findings correspond with the literature data. Therefore, migraine is a significant risk factor for CAD, as this correlation requires further study.

The anterior and posterior cerebral circulations have several clear particularities. Firstly, the amount of blood that flows in the anterior system is bigger with these vessels having a higher caliber, whilst secondly, the location of the vertebral arteries in the vicinity of the subclavian artery and scalene muscle group at the origin and further in course with the cervical vertebrae combined with their relatively small size make them susceptible to injuries related to neck strain either by passive or active movements. Lastly, a carotid artery stroke without viable collateral vessels has a devastating clinical impact with a significantly worse array of symptoms compared to the vertebral counterpart. Therefore, a certain pattern of symptoms is to be expected in the case of carotid artery involvement, such as limb weakness and cortical deficits [21,22,23]. 

Various studies have shown that the carotid arteries are more likely to be affected (regarding incidence) in the case of dissection rather than the vertebral arteries. Simultaneous cervical artery dissections are generally reported to be relatively rare [24,25]. The clinical presentation is variable according to the literature, with various sources suggesting either the fact that neck pain is the most common symptom or a combination of neck pain and limb weakness, with neck pain preceding most of the other acute symptoms [25,26]. Another interesting finding, according to a study, is a stronger association between Horner’s syndrome and carotid artery dissection, whilst further suggesting that patients with Horner’s syndrome at presentation are likely to follow a more benign outcome [27]. Our study has reported the fact that, whilst neck pain is a common symptom, the most incriminated symptoms are limb weakness and cortical deficits, such as aphasia. Another finding within our study was a more common incidence of carotid artery dissection.

The most routinely used diagnosis tools for cervical artery dissections include a set of clinical findings that raise suspicion, followed by imaging studies such as direct angiography, CT angiography (CTA), MR angiography (MRA), and ultrasonographic studies. The most common imaging signs with non-invasive angiographic methods are intimal flap, stenosis, or lumen irregularities, while the distinctive sign with the direct method is an abnormal, false lumen filling. Both the invasive and non-invasive methods have advantages and disadvantages, with the main advantage of the invasive method being the direct periprocedural diagnosis and treatment option [28,29].

Several studies have been performed in this particular regard with interesting findings: non-invasive methods such as CTA and MRA being used simultaneously increase the sensibility and specificity of diagnosis, and the fact that magnetic resonance imaging without angiography failed to diagnose stenosis in up to 20% of cases [30,31,32].

Most of the patients included in our cohort study were diagnosed by non-invasive methods (either MRA or CTA or a combination of both or ultrasonographic studies), while a relative minority of our patients were diagnosed by direct angiography as they also benefitted from direct endovascular treatment on this occasion.

The treatment principles of ischemic stroke associated with CAD are mostly like acute ischemic stroke. The main purpose of the treatment is to limit neurological deficits and long-term morbidity by preventing thromboembolic complications or restoring blood flow. Among the treatment options are thrombolysis or endovascular treatment for eligible patients or a conservative approach (either anticoagulation or antiplatelet therapy) [33].

A relatively large number of studies have been performed regarding the treatment options of patients with CAD. Similar results were achieved either via invasive or non-invasive methods in a study performed on 109 patients with no significant difference between outcomes [33]. Another study managed to suggest the safety of endovascular treatment for patients with recurrent stroke associated with CAD and who initially received preventive antithrombotic therapy with the later occurrence of stroke [34]. A study performed on 44 patients further strengthened the efficacy and safety of endovascular treatment for patients with stroke related to CAD in case of failure of the more conservative methods [35]. The meta-analysis carried out by Bontinis and his collaborators also highlighted the relative safety of the endovascular methods for selected patients with low postprocedural mortality and a statistically significant decrease in morbidity [36].

Our subjects mostly benefited from conservative treatment, meaning either anticoagulant treatment or a combination of antiplatelet drugs and anticoagulant drugs (*n* = 32), while 6 patients benefited from endovascular treatment, and 10 received IV thrombolysis. In the first case, endovascular treatment was attempted following thrombolytic therapy. There were no reported periprocedural or postprocedural complications. Furthermore, the outcomes of the patients appear to be excellent, with a mean Rankin score of 1.85 at discharge. Subsequently, the influence of the acute treatment option on the Rankin score at discharge was studied. Our study did not find any statistically solid evidence that the acute treatment option influenced the outcome of those patients.

There is no clear consensus as to whether the patient who is diagnosed with CAD should receive conservative treatment or acute reperfusion therapy [37]. The current literature manages to highlight several treatment approaches depending on several factors, such as the presence of vessel occlusion, the presence of local compression phenomena, and the presence of stroke syndromes. Therefore, the following treatment approaches are suggested:

- Conservative treatment with antiplatelet drugs for those patients diagnosed with CAD but no objective evidence of stroke, with either no symptoms or only local compression syndromes;
- Conservative treatment but with anticoagulation for patients with evidence of stroke syndromes and vessel occlusion but no eligibility for acute revascularization therapy, and either endovascular treatment or intravenous thrombolysis for those patients presenting within favorable time parameters and obvious debilitating stroke syndromes with confirmed vessel occlusion due to CAD [38].

Compared to the data suggested by the literature and given the fact that most of our patients diagnosed with CAD who received conservative treatment were mostly suffering from associated stroke syndromes, the main majority of patients received anticoagulation therapy right after the diagnosis and upon variable periods of time upon discharge with a minimum of 3 to 6 months and in close relationship with the imagistic results at follow up (in regard to the permeability of the dissected vessel).

Regarding the long-term follow-up, the subjects developing cortical infarctions because of CAD and occlusion of the carotid artery by means of dissection, the most common morbid neurological condition is symptomatic epilepsy. However, those patients have a relatively lower rate of life-threatening seizures compared to those developing seizures after infarctions caused by atherothrombotic occlusion [39,40,41]. In the case of symptomatic seizures associated with CAD, the usage of wider spectrum antiepileptic drugs, including Valproate and Levetiracetam, appears to be a reasonable treatment option, both as a preventive and acute treatment [42].

Moreover, rupture of the vessel wall carries a risk of vasospasm with short-term complications of CAD, quite like those of subarachnoid hemorrhage [43]. These complications should also be treated accordingly as they provide significant morbidity.

The distribution of the population that was included in our study highlights the higher incidence of carotid artery dissections when compared to vertebral artery dissection or multi-vessel dissections (Table 5, Table 6, Table 7 and Table 8). Moreover, the subsequent cerebrovascular event that is associated with the incriminated dissection is also of an increased sevcerity, as shown per NIHSS score at admission in the case of carotid artery dissection. The long-term morbidity of those patients is also influenced by the dissection type. Our study managed to highlight a significantly higher morbidity score for the patients with carotid artery dissection and with multiple vessel dissections when compared to those with vertebral artery dissection. The study elaborated by Lee and his collaborators over a comparable timespan manages to point out the following to comparable results such as:

- The higher incidence of carotid artery dissections;

The relatively small morbidity of the patients and good general outcome of the patients [44].

## 5. Conclusions

Our study managed to point out the following findings regarding the patient suffering a cervical artery dissection:

- Typically, the profile of the patient is a mid-40s Caucasian male;
- The associated relevant risk factors and predisposing conditions are chronic alcohol consumption and hypertension, and there is a significant association between various headache types and the occurrence of CAD;
- The general prognosis of those patients is relatively good, as pointed out by the mean values of the clinical severity scores upon discharge, as well as the mean morbidity score.

CAD associated with stroke or local compression events is a rare cerebrovascular event associated with several important predisposing conditions and with younger age. Most of the patients developed mild quantifiable neurological deficits, with the most common complaint being limb weakness, speech disturbances, and local pain, with a mean NIHSS score at presentation at six points. Our study managed to point out the association between several underlying conditions, such as chronic migraine and hypercholesterolemia, and some modifiable risk factors, such as alcohol consumption, and the clinical severity of the cardiovascular events caused by CAD in the patients with those underlying risk factors. Our results managed to underline a series of findings as follows: the included patients carry an encouraging prognosis with low morbidity scores upon discharge, while the treatment option does not significantly influence the clinical of those patients upon discharge and on follow-ups when possible.

## Figures and Tables

**Table 1:** Correlation between certain risk factors and the gender of the participants.

Characteristic	Men (*n* = 36)	Women (*n* = 18)	Total (*n* = 54)	*p*-Value
Mean age in years	41.6 ± 1.83, IQR = 13	37.7 ± 2.37, IQR = 8	40.32 ± 1.46, IQR = 12	-
Recent history of cervical trauma or neck strain				NS
Yes	12 (33%)	7 (41%)	19 (36%)	
No	24 (67%)	12 (59%)	31 (64%)	
Smoking History				NS
Yes	10 (30.3%)	1 (5%)	11 (22%)	
No	23 (68.7%)	16 (95%)	39 (78%)	
Alcohol consumption				NS
Yes	4 (12%)	2 (11.7%)	6 (12%)	
No	29 (88%)	15 (88.3%)	44 (88%)	
Hypertension				NS
Yes	21 (64%)	9 (52%)	30 (60%)	
No	12 (36%)	8 (48%)	20 (40%)	
Presence of migraine				*p* = 0.006
Yes	5 (15%)	10 (55%)	15 (30%)	
No	28 (85%)	8 (45%)	35 (70%)	
Underlying prothrombotic conditions				NS
Yes	10 (30.3%)	7 (41.2%)	17 (34%)	
No	23 (69.7%)	10 (58.8%)	33 (66%)	
Hypercholesterolemia				NS
Yes	15 (45.4%)	8 (47%)	23 (46%)	
No	18 (54.6%)	9 (53%)	27 (54%)	

The percentages represent the number of subjects from a selected group with the presence of predisposing conditions. The *p*-value of comparison was determined via the chi-square test.

**Table 2:** The affected vascular territories concerning the gender of the patients.

Affected Territories	Male Group	Female Group
Right carotid	11	8
Left Carotid	10	7
Right Vertebral	5	2
Left vertebral	9	2
Multiple arterial dissections	2	1
Presenting symptoms		
Limb weakness	27	13
Speech Disturbances	9	4
Dizziness	4	2
Horner’s syndrome	2	1
Visual disturbances	3	1
Cranial Nerve Palsies	1	1
Diagnosis tools that were used		
DSA	5	1
CT-Angiography	19	8
Doppler Ultrasonography	5	4
MR/ MR Angiography	4	2
Acute treatment options for the patients		
Endovascular	4	2
IV Thrombolysis	7	3
Endovascular and Thrombolytic treatment	4	2
Conservative	25	10

**Table 3:** Shows correlations between certain underlying conditions and the clinical presentation severity score. Comparison values (values of *p*) were obtained either via *t*-test for equal distribution variances or the Mann–Whitney test.

	Mean NIHSS Score with the Mentioned Underlying Condition	Mean NIHSS Score without the Mentioned Underlying Condition	*p*-Value
Alcohol consumption	5.43 ± 0.79, IQR = 15	10.16 ± 2.63, IQR = 8	<0.05
Hypercholesterolemia	10.2 ± 0.93, IQR = 6.5	1.96 ± 0.40, IQR = 11	<0.001
Hypertension	5.2 ± 1.022, IQR = 10	6.53 ± 1.12, IQR = 6	NS
Smoking	5.2 ± 1.022, IQR = 9	6.53 ± 1.12	NS
Migraine presence	5.68 ± 0.91, IQR = 2	7 ± 1.53, IQR = 10	NS
Prothrombotic conditions	6.7 ± 1.41, IQR = 11	5.63 ± 0.95, IQR = 12.5	NS
History of recent neck injury	5.9 ± 0.96	6.29 ± 1.46, IQR = 11	NS

**Table 4:** Comparison of mean Rankin score values according to the gender of the patients. Women appear to have a slightly lower disability score at discharge compared to male patients.

Mean Rankin Score	Male	Female	*p*-Value
	2.3 ± 0.22	1.41 ± 0.17, IQR = 14	0.01

**Table 5:** Comparison of mean Rankin score at discharge regarding the comorbidities of the subjects. Legend: On the left side, the mean Rankin score upon discharge for the patients with the given comorbidities was noted, while on the right side, there is the mean Rankin score at discharge for the patients without the underlying condition. There was no statistically valid correlation when comparing the mean/median values of the Rankin score of the subjects at discharge regarding certain comorbidities or risk factors.

	Mean/Median Value of Rankin Score with the Mentioned Underlying Condition	Mean/Median Value of Rankin Score without the Mentioned Underlying Condition	*p*-Value
Alcohol consumption	3.00 ± 1.72, IQR = −1	2.00± 1.08, IQR = 12	NS
Hypercholesterolemia	3.00 ± 0.93	2.50 ± 0.40	NS
Hypertension	2.00 ± 1.24	1.50 ± 1.13, IQR = 2	NS
Smoking	1.00 ± 1.53, IQR = 13	2.00 ± 1.099	NS
Migraine presence	1.50 ± 0.96, IQR = 14	2.00 ± 1.17	NS
Prothrombotic conditions	2.00 ± 1.33, IQR = 9.5	2.00 ± 1.12, IQR = 1	NS
History of recent neck injury	2.0 ± 1.33, IQR = 4	2.00 ± 1.12, IQR = 1	NS

**Table 6:** Comparison of mean/median values of the Rankin and NIHSS scores concerning the chosen acute treatment option. There was no statistically relevant difference between the Rankin scores at the discharge of the patients receiving acute treatment compared to the patients receiving conservative treatment.

Acute Treatment That Was Performed	Thrombolysis	Thrombectomy	Conservative Treatment	*p*-Value
Mean/Median Rankin score at discharge	2	2	1	NS

**Table 7:** The presence of certain comorbidities and the vessel that was affected by dissection.

	Carotid Artery Dissection	Vertebral Artery Dissection	Multiple Dissections
Alcohol consumption	3	2	2
Hypercholesterolemia	21	7	2
Hypertension	11	5	0
Smoking	8	6	1
Migraine presence	8	4	1
Prothrombotic conditions	2	4	1
History of recent neck injury	11	6	1

**Table 8:** The mean NIHSS score at admission and the mean Rankin score upon discharge based on the vessel that was affected by dissection. The mean NIHSS score of the patients with carotid artery dissection is significantly higher than the other subgroups, while the patients with multiple dissections have a significantly higher morbidity score upon discharge.

	Vertebral Artery Dissection	Carotid Artery Dissection	Multiple Dissection	*p*-Value
Mean NIHSS score upon presentation	0.5 ± 0.73, IQR = −3	8.52 ± 4.89	4 ± 2	<0.01
Mean Rankin score at discharge	1 ± 1	2 ± 1.5	2.5 ± 0.5	<0.01

## Data Availability

Data is available from the corresponding author on reasonable request.

## Abbreviations

CAD	cervical artery dissection
CTA	computer tomography angiography
CT	computer Tomography
MR	magnetic resonance
MRA	magnetic resonance angiography
NIHSS	National Institute of Health Stroke Scale
mRS	modified Rankin scale
DSA	direct subtraction angiography
